# A season for indulgence

**DOI:** 10.1017/ehs.2024.49

**Published:** 2025-01-20

**Authors:** Ruth Mace

**Affiliations:** Department of Anthropology, UCL, London, UK

First, I must apologise to those of you that may have experienced some delays in publishing your papers over the summer. As you probably heard from Cambridge University Press, they were subjected to a cyber attack which impacted all their journals (including this one). Fortunately, the *EHS* submission and peer-review processes continued as usual throughout, and the production systems are now restored and back on track; hopefully all your papers are now coming through.

We said goodbye to Dietz Stout as an assistant editor, who is now becoming head of his department, after many years of great service to *EHS* for which I am very grateful; we will miss him. We have also recently welcomed as new assistant editors Pat Barclay, an evolutionary psychologist from Guelph (Canada), Chris Venditti, a comparative evolutionary biologists and paleoanthropologist from Reading (UK), and Caroline Schuppli, a primatologist from Max Planck (Germany), who will start in January. As you can see, we are still striving to cover as wide as possible a range of topics within the evolutionary human sciences. Thank you as ever to all the assistant editors working for *EHS*, and all of you who submit your work and those of you who review it, to help make it better and easier to understand.

Our special collection on Causal Inference was finally born, along with a webinar in which some of the contributors described their papers (which you can see on the website). Other special collections in the works include forthcoming ones on Researching Sensitive Topics, Cultural Evolution of the Arts, Scientific Racism, and possibly a few more that are currently only in an embryonic state. All are still open for contributions if you have some research to report on these topics.

One of the highlights of my year was doing a stage performance in a public theatre – well, when I say performance, I was just sitting on a sofa being interviewed about the evolution of behaviour, along with a proper singer-songwriter who actually did a performance (as part of the Sophia Club Live Philosophy series). It was very gratifying to discover that the general public of Hoxton in London are interested in our field, or at least interested enough to buy a cheap ticket and spend the evening in one of the last remaining local Victorian music halls in London, listening to us chat about evolutionary anthropology. I have given scores of academic talks, many that are in public lectures, but this was a new format for me. It is sometimes hard to justify studying something as esoteric as human evolution, when there are so many serious and pressing problems in the world. Maybe some of our work can speak directly to understanding some of those issues, and maybe some of it cannot. The UK university sector appears not to be entirely convinced about evolutionary human sciences, based on the recent closure of several anthropology and archaeology departments. However, it is not just social science for the chop – universities are closing other departments too – and the UK university funding system appears to be getting tougher. But I do truly believe that we need to preserve corners in the world for science and thought that are not just about money. I can only argue that it is a benefit to civilization for at least some of us to research, teach and learn about the science of why we are the way we are, and I still hope all countries can continue to find whatever space they can afford to do just that. Both scientists and the public are very happy to enter such spaces when given the chance.

Have a very happy Christmas holiday. Remember that children have evolved to manipulate their parents. Enjoy being manipulated by your children or grandchildren if you have any. And enjoy not being manipulated if you do not.

